# Unraveling the Role of PET in Cervical Cancer: Review of Current Applications and Future Horizons

**DOI:** 10.3390/jimaging11020063

**Published:** 2025-02-17

**Authors:** Divya Yadav, Elisabeth O’Dwyer, Matthew Agee, Silvina P. Dutruel, Sonia Mahajan, Sandra Huicochea Castellanos

**Affiliations:** 1 Molecular Imaging & Therapeutics, Department of Radiology, Weill Cornell Medicine, New York, NY 10065, USA; 2 Molecular Imaging & Therapeutics, Department of Radiology, Memorial Sloan Kettering Cancer Center, New York, NY 10065, USA

**Keywords:** PET, PET/CT, cervical cancer, staging, restaging, response assessment

## Abstract

FDG PET/CT provides complementary metabolic information with greater sensitivity and specificity than conventional imaging modalities for evaluating local recurrence, nodal, and distant metastases in patients with cervical cancer. PET/CT can also be used in radiation treatment planning, which is the mainstay of treatment. With the implementation of various oncological guidelines, FDG PET/CT has been utilized more frequently in patient management and prognostication. Newer PET tracers targeting the tumor microenvironment offer valuable biologic insights to elucidate the mechanism of treatment resistance and tumor aggressiveness and identify the high-risk patients. Artificial intelligence and machine learning approaches have been utilized more recently in metastatic disease detection, response assessment, and prognostication of cervical cancer.

## 1. Introduction

Cervical cancer is the fourth most common cancer in women globally, with a new case rate of 7.6 and a death rate of 2.2 per 100,000 women per year [1]. The highest rates of cervical cancer incidence and mortality are in low- and middle-income countries [2]. In developed countries, active screening with the Papanicolaou (Pap) smear test allows early detection of preclinical and early-stage cervical cancer [3,4]. Women with precancerous lesions and early cervical lesions typically remain asymptomatic. In invasive cervical cancers and more advanced stages, symptoms may include abnormal vaginal bleeding (such as postcoital bleeding, intermenstrual bleeding, menorrhagia, spotting between periods, and postmenopausal bleeding), abnormal discharge, painful intercourse, and bleeding following vaginal examination. In some cases of advanced-stage cancer, symptoms of bowel or urinary tract obstruction may also be present at the time of diagnosis [5]. The 2021 update of the International Federation of Gynecology and Obstetrics (FIGO) guidelines allowed the use of positron emission tomography/computed tomography (PET/CT) and other modalities such as computed tomography (CT) and magnetic resonance imaging (MRI) to provide anatomic and metabolic information in cervical cancer staging, which can impact patient management [6,7]. FIGO guidelines suggest the use of these imaging modalities for measurement of the primary tumor size, for assessment of extension into the surrounding tissues and adjacent organs, and for assessment of location and characteristics of the retroperitoneal lymph nodes [6]. The National Comprehensive Cancer Network (NCCN) guidelines recommend the use of [^18^F]Fluoro-2-deoxy-glucose (FDG) PET/CT in several specific scenarios. It is highly suggested for high-risk, locally advanced cervical cancer in Stage IB2 or higher. PET/CT is also recommended for the detection of distant metastasis in situations where conventional imaging has unclear findings. Additionally, it is useful for the evaluation of recurrence when clinical symptoms, pelvic examination, or other imaging findings raise suspicion. Finally, PET/CT is valuable in radiation treatment planning, especially in cases with extensive nodal involvement or uncertain tumor boundaries [8]. Accurate staging using the FIGO staging system (Table 1) is essential for cervical cancer management. Surgical approaches such as radical hysterectomy or fertility-preserving trachelectomy are critical in early-stage (Stage IA, IB1) cervical cancer. Pelvic lymphadenectomy is often performed alongside surgery to assess the extent of disease spread and in cases of nodal metastases. Postoperative chemoradiation is recommended in high-risk cases to reduce the risk of recurrence. Radiation therapy or chemoradiation remains a cornerstone of treatment, particularly for locally advanced stages (IB2 and beyond) and non-surgical candidates. For metastatic or recurrent cervical cancer, chemotherapy and newer therapies such as targeted therapy and immunotherapy can be utilized as treatment options [9]. PET imaging serves as an invaluable tool in patient management by providing accurate staging, which is necessary for personalized treatment planning. We aim to focus on discussing the current role and future perspectives of the utility of PET imaging in the management of patients with cervical cancer.

## 2. FDG PET/CT in Cervical Cancer

### 2.1. FDG PET in Staging

The increased glucose metabolism in cervical cancer cells leads to increased FDG uptake through upregulated glucose transporters (GLUT-1). Primary cervical cancer accumulates FDG with higher uptakes in squamous cell histology (maximal standardized uptake value {SUVmax} average of 11.91, range 2.50–50.39) than in non-squamous histology (average SUVmax of 8.05, range 2.83–13.92). The mean SUVmax was even higher for poorly differentiated than well-differentiated cervical tumors (*p* = 0.047) [10]. FDG PET/CT has a limited role in early-stage cervical cancer (FIGO ≤ IB) due to low sensitivity in the detection of subcentimeter lesions because of low spatial resolution, making it difficult to delineate local spread, for example, parametrial extension and vaginal involvement, which is optimally demonstrated by MRI. Also, it cannot reliably differentiate between post-cone biopsy or loop electrosurgical excision procedure (LEEP) inflammatory change and viable tumor [11]. There is also a limited role of PET in staging clinically early-stage disease, since the prevalence of nodal disease in this group is low, and, if present, it is often small-volume micrometastases below the resolution of PET [12].

**Figure 1:**
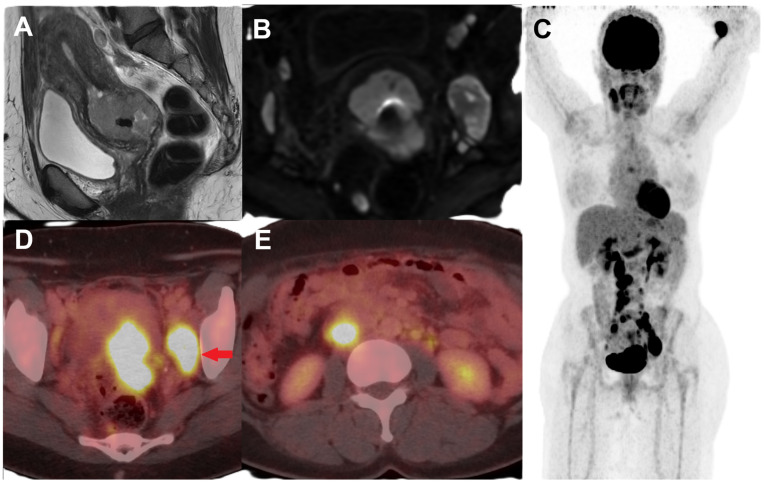
Female with newly diagnosed HPV-associated squamous cell cervical cancer, Stage IIIC. T2-weighted sagittal MR (**A**) and diffusion-weighted (**B**) images show a circumferential mass involving the cervix, extending into the lower uterine segment, and suspicious parametrial invasion in the left posterolateral region as well as enlarged pelvic lymph nodes. (**C**) Maximum intensity projection (MIP) image of FDG PET/CT performed for initial staging showing FDG-avid primary cervical lesion with metastatic FDG-avid enlarged left obturator node ((**D**), red arrow) and FDG-avid retroperitoneal metastatic lymph nodes (**E**).

FDG PET/CT has an established role in the initial staging of locally advanced cervical cancers (LACC) (FIGO stage ≥ IB), particularly in the evaluation of nodal disease (Figure 1) and distant metastases. The detection of nodal metastases is essential for optimal treatment planning, which is relatively common in patients with LACC at diagnosis. PET/CT has a higher accuracy in the detection of pelvic and para-aortic lymph node metastases in LACC with pooled sensitivity and specificity of 72% and 96%, respectively [13]. The positive and negative predictive values for pelvic lymph nodes with PET/CT were 0.96 and 0.81, respectively, and were 0.86 and 0.61 for para-aortic nodes, respectively [14]. For same FIGO stage and treatment groups of patients, the patients with FDG-positive nodes have worse prognosis than those with FDG-negative nodes, which suggests that FDG-positive nodes require personalized treatment intensification for improved progression free survival [15].

False-positive findings on FDG PET can occur most commonly in lymph nodes, particularly in areas like the axilla and groin, which can be avid due to reactive etiology and may be mistaken for metastatic disease, prompting further investigation. Additionally, the limitations of FDG PET include false-negative results due to low spatial resolution, which can miss micrometastases, as well as false-positive results caused by bowel uptake or urinary artifacts [16]. Distant metastatic disease can be detected by FDG PET/CT in patients with cervical cancer at the time of initial diagnosis, commonly spreading to lungs, bones, and liver through hematogenous spread [17]. PET/CT can detect distant metastases with high diagnostic accuracy with reported sensitivity, specificity, positive predictive value, and negative predictive value of 54.8%, 97.7%, 79.3%, and 93.1%, respectively [18].

The FDG avidity quantified as SUVmax of the primary cervical tumor on baseline PET is predictive of treatment response and disease outcome [19]. Additionally, volume-based metabolic parameters including metabolic tumor volume (MTV) and total lesion glycolysis (TLG) are also proven to be significant independent prognostic factors. MTV and TLG were significantly higher (*p* = 0.0006 and *p* = 0.03) in patients with positive lymph node metastases [20]. The number and site of positive pelvic lymph nodes on pretreatment PET/CT are also independent prognostic factors for recurrence of disease in patients with LACC and overall survival [21]. The PET-based para-aortic nodal involvement remains the strongest predictor of survival in a multivariate logistic regression analysis [22]. Thus, these quantitative PET parameters and PET-determined nodal status can aid in selecting patients who could benefit from therapeutic optimization and closer surveillance.

### 2.2. FDG PET in Recurrence

**Figure 2:**
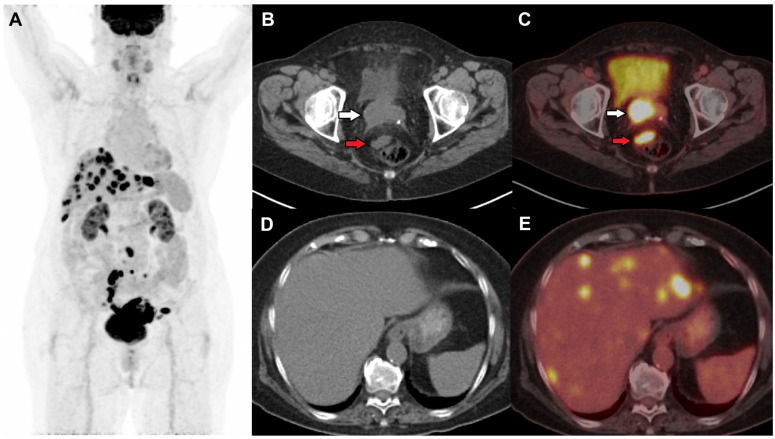
(**A**) Restaging FDG PET/CT was performed in a female with initial stage 1B2 status post-hysterectomy and bilateral salpingo-oopherectomy and suspected recurrence on local examination and vaginal bleeding 18 months after the surgery. Axial CT and fused PET/CT images show an FDG-avid soft tissue nodule in the right vaginal cuff (white arrow) suspicious of local recurrence and an FDG-avid perirectal node (red arrow) (**B**,**C**), and multiple FDG-avid hepatic metastases (**D**,**E**).

The role of PET/CT in cervical cancer is crucial in the assessment of recurrent disease [16]. Disease recurrence within two years is high in patients with LACC after treatment completion. Common sites of recurrence include the vaginal vault, cervix (after chemoradiation), pelvic sidewalls, and distant sites such as lungs, liver, and bones. An example of cervical cancer recurrence post-total abdominal hysterectomy is shown in Figure 2 with local recurrence in the vaginal cuff (Figure 2C), pelvic nodes, and liver metastases (Figure 2E). A meta-analysis by Chu et al. described the pooled sensitivity and specificity values of 82% and 98%, respectively, for identifying locoregional recurrence by FDG PET/CT in recurrent cervical cancer patients [23]. PET/CT has been shown to have higher sensitivity in the detection of suspected recurrent cervical cancer after radiotherapy than MRI (97.6% vs. 80.1%) [24]. FDG PET was also found to be significantly superior to CT or MRI (sensitivity: 92% vs. 60%; AUC: 0.962 vs. 0.771; *p* < 0.0001) in identifying metastatic lesions [25]. For locoregional and metastatic disease detection in patients with suspected recurrence, the pooled sensitivity of PET and PET/CT was 97%, and when combined with elevated tumor markers, the pooled sensitivity was even higher at 99% [26]. PET/CT findings have been responsible for treatment modifications in 57% of the patients [26].

### 2.3. FDG PET in Response Assessment

**Figure 3:**
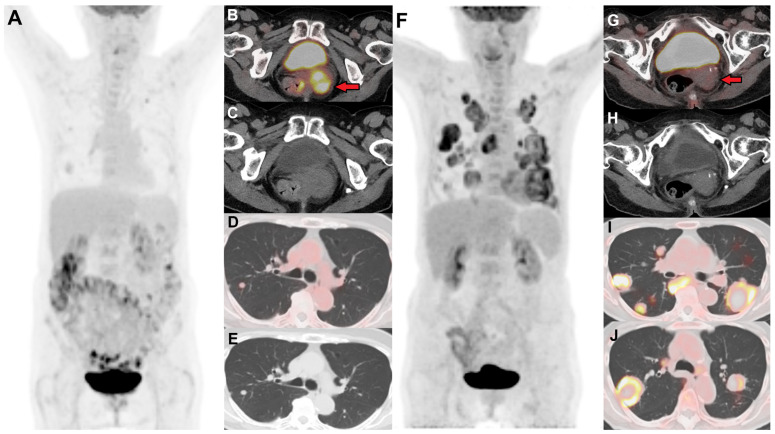
(**A**) Baseline FDG PET/CT in a female with stage IVB cervical cancer showing an FDG-avid primary cervical lesion ((**B**) (red arrow), (**C**)), pelvic nodes, and a few lung nodules suspicious for metastases (**D**,**E**). (**F**) Follow-up PET/CT 12 months post-chemoradiation shows resolution of FDG avidity in the cervix ((**G**) (red arrow), (**H**)), consistent with post-radiation treatment changes. However, there are new and increased sizes of numerous FDG-avid bilateral pulmonary metastases (**I**,**J**), with some lesions showing central necrosis.

It is now common practice to perform FDG PET/CT three months post-completion of chemoradiotherapy in LACC and metastatic disease as recommended by NCCN guidelines [8]. It can predict treatment outcomes and is often used to tailor management, including adjuvant therapy and follow-up. FDG avidity can help differentiate between complete metabolic response versus metabolically active residual disease. Figure 3 demonstrates complete metabolic response in the cervix on post-treatment follow-up PET/CT; however, there is progression of disease with increased FDG-avid pulmonary metastases. PET/CT can assess the treatment response to chemoradiation with higher diagnostic sensitivity and specificity for the detection of tumor metastases (97% and 99%, respectively) [27]. PET/CT can aid in the early identification of non-responders to decrease treatment failures and avoid the toxicity of futile treatment. Patients with persistent positive FDG uptake after chemoradiation on three months post-treatment PET/CT had shorter progression-free survival (PFS) than those with negative results. A multivariate analysis demonstrated that patients with positive FDG PET/CT results had almost nine times higher risk of progression (*p* < 0.001) [24]. A complete metabolic response has been associated with an excellent survival outcome, with a 3-year cause-specific survival rate of 100%. In contrast, a partial metabolic response is associated with an intermediate survival outcome, showing a 3-year cause-specific survival rate of 51%. Progression detected on post-therapy PET/CT, however, corresponds to a very poor survival outcome, with a 3-year cause-specific survival rate of only 17%. Thus, FDG response to therapy serves as a reliable predictor of patient survival outcomes [22].

### 2.4. FDG PET in Radiation Treatment Planning

**Figure 4:**
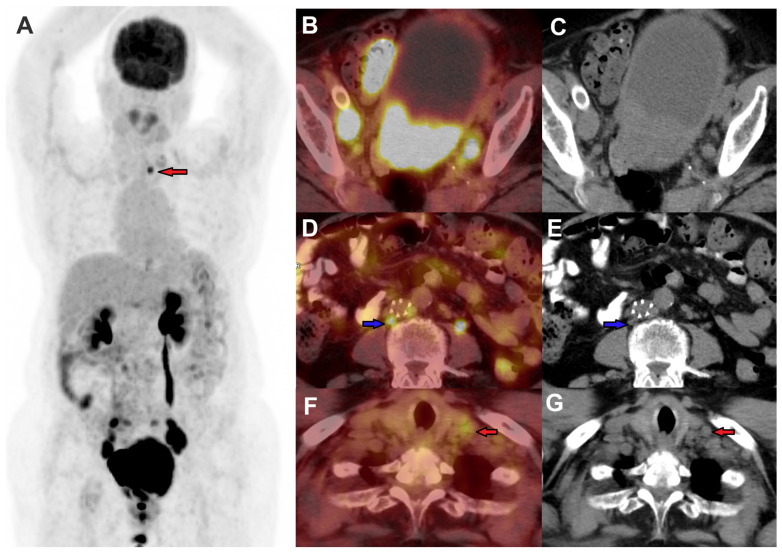
FDG PET/CT was performed in a female for a stage IVB cervical cancer staging, MIP image (**A**), axial fused PET/CT, and CT images demonstrating FDG-avid primary cervical lesions, with FDG-avid metastases to bilateral pelvic nodes (**B**,**C**), retroperitoneal nodes (blue arrow) (**D**,**E**), and a small left supraclavicular node (red arrows) (**F**,**G**).

**Figure 5:**
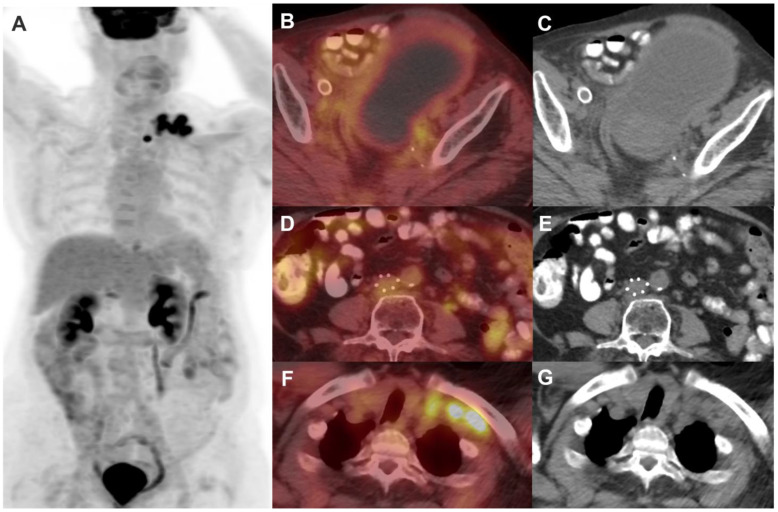
The same patient shown in Figure 4 underwent post-treatment follow-up FDG PET/CT 3 months after concurrent chemotherapy and external beam radiation to the pelvis and retroperitoneum. Maximum Intensity Projection image of FDG PET/CT (**A**) demonstrates a cluster of metabolically active left supraclavicular lymph nodes. There is resolution of FDG-avid primary cervical lesion, pelvic nodes (**B**,**C**), and retroperitoneal nodes (**D**,**E**). There is, however, an increase in the number and size of FDG-avid left supraclavicular lymph nodes (**F**,**G**), suggestive of disease progression outside the radiation treatment field.

PET/CT has become a standard part of pre-radiation treatment planning in patients with LACC. PET/CT can alter the radiotherapy treatment plan in approximately 10–20% of the patients by detecting extra-pelvic nodal metastases [15,28]. This can be achieved by either extending the treatment field or applying an additional radiotherapy dose (boost) to metastatic lymph nodes, resulting in better survival. This is particularly important in the detection of paraaortic and supradiaphragmatic nodal metastases [29]. Furthermore, PET-guided radiation techniques such as intensity-modulated radiation therapy (IMRT) have been shown to improve local control rates as well as clinical outcomes by delivering higher doses of radiation to the target while minimizing treatment-related toxicity [30]. Figure 4 demonstrates a baseline FDG PET/CT imaging in a patient with cervical cancer, where FDG PET/CT detected FDG-avid retroperitoneal lymph node metastases and a small supraclavicular node, in addition to primary cervical lesion and pelvic lymph nodes. Concurrent systemic chemotherapy and external beam radiation was planned to include pelvic and retroperitoneal nodes guided by PET/CT findings. On follow-up FDG PET/CT as shown in Figure 5, there is complete metabolic resolution of primary cervical lesion, pelvic as well as retroperitoneal nodes. There are new metabolically active left supraclavicular lymph nodes which reflect progression outside the radiation treatment field, subsequently confirmed to be metastatic in cytology. FDG PET/CT can impact clinical treatment decisions and guide patient management strategies.

## 3. FDG PET/MR in Cervical Cancer

FDG PET/MRI is a hybrid imaging technique with simultaneous or sequential acquisition of MRI and PET images. Newer scanners have overcome the initially faced misregistration issues and utilize MRI-based attenuation-correction techniques, making these more feasible for practical use. PET/MR can overcome the technical challenges of lower soft tissue resolution often witnessed with PET/CT, particularly in the evaluation of local extension and pelvic surgical bed. High spatial resolution small field-of-view (FOV) T2-weighted pelvic MR images without fat suppression improve the diagnostic accuracy of hybrid PET/MR imaging for the evaluation of local tumor extent in determining the primary T stage in cervical cancer [31]. PET/MRI demonstrated the highest diagnostic accuracy for cervical cancer (94.90%) compared to PET/CT, MRI, and CT (83.67%, 75.51%, and 69.39%, respectively) (*p* < 0.05). Additionally, PET/MRI exhibited a higher detection rate for local invasion in pelvic organs, including vaginal, uterine, bladder, and cervix, compared to PET/CT, MRI, and CT (*p* < 0.05) [32]. The combined sensitivity and specificity of FDG PET/MRI for detecting cervical cancer lymph node metastasis were 85% and 94%, respectively [33]. The multiplanar imaging of MRI provides high soft tissue contrast in local staging with metabolic information from PET for whole body increases diagnostic accuracy for nodal staging and assessment of distant metastasis and tumor recurrence. Additionally, PET/MRI can serve as a functional imaging platform, combining quantitative imaging biomarkers from both PET and functional multiparametric MRI techniques. These biomarkers can be used to evaluate treatment response and have demonstrated prognostic significance [34]. However, due to limited availability, FDG PET/MRI is not yet the standard of care in most institutions.

## 4. Limitations of FDG and Need for Newer Radiotracers

Thus far, we have discussed how FDG PET is an essential tool for evaluating cervical cancer, particularly in detecting tumor recurrence and accurate staging, but it does have some limitations. These include the potential for false positives, such as reactive lymph nodes, as well as false negatives often encountered in early-stage or small tumors and certain tumor types. Well-differentiated squamous and non-squamous cell carcinomas, such as adenocarcinomas, neuroendocrine tumors, and cervical sarcomas, demonstrate lower FDG activity [10]. While FDG PET provides valuable metabolic information, it remains a non-specific radiotracer and can sometimes be challenging to differentiate from benign lesions or inflammation such as cervicitis [35]. These limitations and tumor heterogeneity created the need for investigating alternative radiotracers that target specific molecular pathways involved in cancer growth and metastasis. These novel PET tracers aim to improve tumor detection and enhance tumor characterization by providing more accurate insights into tumor microenvironments. Newer PET tracers, which can target hypoxia and tumor proliferation markers, hold the potential to overcome these limitations and may also serve as therapeutic targets.

## 5. Hypoxia PET Imaging in Cervical Cancer

Intra-tumoral hypoxia has been investigated in cervical cancer as a poor prognostication marker, often demonstrating poor response to treatment. It is associated with more aggressive phenotypes with an increased likelihood of local recurrence and distant metastases. Hypoxia is a major limiting factor in response to radiation therapy, and hypoxia-modifying strategies such as radiosensitizers and normobaric or hyperbaric oxygen have been shown to improve radiotherapy outcomes. Modern approaches that combine radiation with molecular targeted drugs or escalate the radiation dose to hypoxic tumor regions rely heavily on robust hypoxia biomarkers. Tumor hypoxia imaging with PET tracers such as [^18^F]FMISO (fluoromisonidazole), [^18^F]FAZA (fluoroazomycin–arabinoside), and [^64^Cu]Cu(ATSM) {diacetyl-bis(N4-methylthiosemicarbazone)} allows for the detection of hypoxic regions, which serve as a biomarker of poor prognosis in patients with cervical cancer [36,37]. These PET tracers can provide valuable information about tumor oxygenation by visualization and quantification of hypoxic areas non-invasively. Among the nitroimidazoles, [^18^F]FMISO has been investigated in-depth in cervical cancer as well as other solid tumors and has been shown to identify hypoxic tumor subvolumes and intratumoral heterogeneity. Combined use of FDG and [^18^F]FMISO multiparametric PET/MRI hybrid imaging demonstrated the viability of this modality in cervical cancer cases in sixteen patients with histologically proven LACC [38].

Tumoral hypoxic volume can be identified in the majority of cervical tumors (89% when using tumor/muscle or tumor/blood pool > 1.2 as a threshold) on the 2-h [^18^F]FAZA imaging. [^18^F]FAZA shows higher tumor-to-background accumulation as compared to [^18^F]FMISO. The extent of hypoxia has been shown to vary markedly between tumors but not significantly with different reference tissues/thresholds on pre-chemoradiation 18F-FAZA PET/CT [39]. Tumor [^60^Cu][Cu(ATSM)] uptake was inversely related to progression-free survival and cause-specific survival (*p* = 0.006 and *p* = 0.04, respectively). A threshold of tumor-to-muscle (T/M) ratio of 3.5 detected patients who were at risk of disease recurrence; the 3-y progression-free survival of patients with normoxic tumors (as defined by T/M of < or = 3.5) was 71%, and that of patients with hypoxic tumors (T/M of >3.5) was 28% (*p* = 0.01) [40]. [^64^Cu][Cu(ATSM)] can produce better quality images with lower noise and a similar magnitude of tumor uptake as compared to [^60^Cu][Cu(ATSM)] in cervical cancer patients [41]. In the context of cervical cancer, hypoxia PET imaging can be used in identifying high-risk patients, guiding targeted therapies, and monitoring tumor response to treatment.

## 6. Fibroblast Activation Protein Inhibitor (FAPI) PET Imaging in Cervical Cancer

FAPI PET imaging is an emerging molecular imaging technique that targets fibroblast activation protein (FAP), a protein expressed on the surface of cancer-associated fibroblasts (CAF) in various tumors [42,43]. It specifically targets and binds to FAP-expressing fibroblasts in the tumor stroma, often associated with aggressive growth and metastasis. Recently, the [^68^Ga]Ga-FAPI PET tracer has demonstrated promising results in providing information about tumor microenvironments in gynecologic malignancies. [^68^Ga]Ga-FAPI PET/CT (including both FAPI-04 and FAPI-46) has been shown to localize in gynecological malignancies with high tumor-to-background uptake [44]. [^68^Ga]Ga-FAPI-46 PET/CT has also shown a higher detection rate for lymph nodes and distant metastases than [^18^F]FDG PET/CT, especially in the detection of the liver and osseous metastases [45,46]. [^68^Ga]Ga-FAPI-46 PET uptake and tumor volume have been correlated with HIF-1 (hypoxia-inducible factor) expression, which has been suggested as a surrogate marker of hypoxia as well [47].

## 7. Chemokine CXCR4-Directed PET Imaging in Cervical Cancer

[^68^Ga]Ga-Pentixafor targets CXC chemokine receptor 4 (CXCR4), which is overexpressed in various solid cancers [48]. The CXCL12-CXCR4 signaling axis is crucial not only for tumor progression and metastasis but also for the treatment-induced recruitment of CXCR4-expressing cytotoxic immune cells [49]. The feasibility of imaging CXCR4 expression using [^68^Ga]Ga-Pentixafor PET/CT in cervical cancer has been investigated and correlated with immunohistochemistry stains of cervical cancer tissue samples. CXCR4 expression was correlated with more aggressive histology, lymph node metastasis, and resistance to chemoradiation [50]. Although currently there is not enough evidence demonstrating the role of [^68^Ga]Ga-Pentixafor in cervical cancer, CXCR4-PET imaging could potentially be used for prognostication and patient selection for CXCR4-targeting therapies.

## 8. Future Directives

The radiomic parameters, such as first-order statistical and second-order texture analysis on the basis of the Gray-Level Co-occurrence Matrix (GLCM), can provide additional information to predict treatment outcomes in cervical cancer patients [51]. The light GBM-based PET radiomics model showed potential to predict the histological subtypes of locally advanced cervical cancer, differentiating cervical adenocarcinoma and squamous cell carcinoma, and may serve as a promising noninvasive approach for the diagnosis of cervical cancer [52]. PET-based metabolic and volumetric parameters can provide valuable insights into intratumoral heterogeneity of FDG uptake on pretreatment FDG PET/CT. SUV and a few PET radiomic features were significantly different between pathologic responders and nonresponders [53]. Deep learning models have also been developed for assessing FDG PET/CT for early prediction of local and distant failures for patients with locally advanced cervical cancer [54]. Most studies combined clinical, imaging, and metabolic parameters to predict survival with greater accuracy in training machine learning models [55]. This advanced imaging analysis, when combined with artificial intelligence, paves the way for personalized treatment strategies, ultimately improving patient care and survival rates. However, there is a need for large, prospective, multicenter studies with standardized protocols to validate the use of radiomics and machine learning techniques before they can be applied for clinical use for prognostication and patient management in cervical cancer.

## 9. Conclusions

The use of FDG PET/CT imaging in the management of cervical cancer represents a significant advancement in personalized medicine. FDG PET can aid in accurately staging LACC, identifying recurrence, and assessing treatment response. Incorporation of metabolic information obtained from FDG PET enables tailoring of radiation treatment planning, which can impact clinical outcomes. Utilizing metabolic information in conjunction with tumor markers can help in the prognostication of cervical cancer. The multidisciplinary approach utilizing FDG PET/CT and PET/MR or MR in cervical cancer management contributes to improved patient outcomes and quality of life. However, FDG PET has shown some limitations, which have driven the search for alternative PET tracers that may enhance tumor detection, improve characterization, and even serve as therapeutic targets. Additionally, radiomic analysis has shown promise in distinguishing histological subtypes and predicting treatment outcomes. While these advancements pave the way for personalized treatment, large, prospective, multicenter studies with standardized protocols are essential for clinical validation.

## Figures and Tables

**Table 1:** FIGO staging of cervical (cervix uteri) cancer (2018).

Stage	Description
I	The carcinoma is strictly confined to the cervix
IA	Invasive carcinoma that can be diagnosed only by microscopy, maximum depth of invasion ≤ 5 mm
IA1	Measured stromal invasion ≤ 3 mm in depth
IA2	Measured stromal invasion > 3 and ≤5 mm in depth
IB	Invasive carcinoma with measured deepest invasion > 5 mm (greater than Stage IA); lesion limited to the cervix uteri with size measured by maximum tumor diameter
IB1	Invasive carcinoma > 5 mm depth of stromal invasion and ≤2 cm in greatest dimension
IB2	Invasive carcinoma > 2 and ≤4 cm in greatest dimension
IB3	Invasive carcinoma > 4 cm in greatest dimension
II	The carcinoma invades beyond the uterus but has not extended onto the lower third of the vagina or to the pelvic wall
IIA	Involvement limited to the upper two-thirds of the vagina without parametrial involvement
IIA1	Invasive carcinoma ≤ 4 cm in greatest dimension
IIA2	Invasive carcinoma > 4 cm in greatest dimension
IIB	With parametrial involvement but not up to the pelvic wall
III	The carcinoma involves the lower third of the vagina and/or extends to the pelvic wall and/or causes hydronephrosis or nonfunctioning kidney and/or involves pelvic and/or para-aortic lymph nodes
IIIA	The carcinoma involves the lower third of the vagina, with no extension to the pelvic wall
IIIB	Extension to the pelvic wall and/or hydronephrosis or nonfunctioning kidney
IIIC	Involvement of pelvic and/or para-aortic lymph nodes, irrespective of tumor size and extent
IIIC1	Pelvic lymph node metastasis
IIIC2	Para-aortic lymph node metastasis
IV	The carcinoma has extended beyond the true pelvis or biopsy-proven involvement of the mucosa of the bladder or rectum
IVA	Spread of the growth to adjacent pelvic organs
IVB	Spread to distant organs

## Data Availability

No new data were created.
